# Endolichenic fungi: the lesser known fungal associates of lichens

**DOI:** 10.1080/21501203.2017.1352048

**Published:** 2017-07-17

**Authors:** Trichur S. Suryanarayanan, Nagamani Thirunavukkarasu

**Affiliations:** a Vivekananda Institute of Tropical Mycology, Ramakrishna Mission Vidyapith, Chennai, India; b PG & Research department of Botany, Ramakrishna Mission Vivekananda College, Chennai, India

**Keywords:** Endophyte, lichen symbiont, lichen microbiome

## Abstract

Lichens are the result of a stable mutualism between a fungal and a photosynthesising partner (alga or cyanobacterium). In addition to the fungal partner in this mutualism, lichens are associated with endolichenic fungi which reside inside their thalli. The endolichenic fungi appear to have evolved with the lichen and many of them are a source of novel metabolites vested with unique bioactivities. There is very little information on the biology of endolichenic fungi and their interactions with the other components of a lichen microbiome. There is an urgent need to understand these aspects of endolichenic fungi such that their ecology and economic potential are known more completely. The current knowledge on endolichenic fungi is reviewed here.

## Introduction

Exploring less-studied ecosystems and habitats for microbes including fungi is a profitable endeavour both in terms of understanding their biology and exploiting their novel genes for technology. Lichens, by supporting many different microbes and exhibiting multipartite interactions between them, represent a miniature ecosystem worthy of such exploration. We underscore this by highlighting the need to study the endolichenic fungi – one of the different groups of microbes associated with lichens.

### Endolichenic fungi

A lichen is “an ecologically obligate, stable mutualism between an exhabitant fungal partner (the mycobiont) and inhabitant population of extracellularly located unicellular or filamentous algal or cyanobacterial cells (the photobiont)” (Hawksworth and Honegger 1994). Although the mycobiont in most lichens is an ascomycete, a recent study shows that a basidiomycete yeast is invariably involved in this mutualism as a third partner (Spribille et al. 2016). Additionally, lichens are associated with fungi lichenicolous fungi, endolichenic fungi and culturable and non-culturable non-photosynthetic bacteria (Biosca et al. 2016; Muggia et al. 2016). The lichens along with their associates represent a successful mode of symbiosis as they have existed for over 600 million years (Yuan et al. 2005) and currently dominate nearly 10% of the Earth’s terrestrial ecosystems (Papazi et al. 2015).

The endolichenic fungi are akin to the endophytic fungi of vascular plants in many aspects; they occur internally in the lichens, do not produce any visible disease symptoms and are transmitted horizontally (Arnold et al. 2009; Kannangara et al. 2009; U’Ren et al. 2012). Furthermore, like the endophytic fungi, they produce an array of secondary metabolites such as alkaloids, quinones, furanones, pyrones, benzopyranoids, xanthones, terpenes, steroids, peptides and allycylic compounds (Paranagama et al. 2007; He et al. 2012; Yang et al. 2012, 2016; Li et al. 2015; Samanthi et al. 2015; Kellogg and Raja 2016; Yuan et al. 2016). These metabolites exhibit many novel bioactivities including antibacterial, antifungal, cytotoxic and antioxidant activities (Kellogg and Raja 2016; Suryanarayanan et al. 2017). All lichens, from the Arctic to the tropics, studied for their endolichenic fungi have been shown to harbour these fungi (Arnold et al. 2009; Suryanarayanan et al. 2005, 2017; Li et al. 2007; Kannangara et al. 2009; Tripathi and Joshi 2015) (Table 1). As only a tiny fraction of the estimated 18,500 lichen species (Nash 2008) has been studied for their endolichenic fungal assemblages, information regarding this ecological group of fungi is limited (Tripathi et al. 2014). Most of the literature on endolichenic fungi pertains to their ability to produce novel secondary metabolites vested with some biological activity (Kellogg and Raja 2016). It is essential to know more about the biology of endolichenic fungi to understand their role in the establishment and maintenance of the lichen symbiosis and their interactions with the lichen microbiome.

**Table 1.** List of lichens studied for their endolichenic fungi.

Lichen	Location	Reference
*Cladonia* sp.		Petrini et al. (1990)
*Stereocaulon* sp.		
*Parmelia taractica*	–	Girlanda et al. (1997)
*Peltigera praetextata*		
*Dirinaria picta*	Chennai, India	Suryanarayanan et al. (2005)
*Heterodermia diademata*		
*Physcia aipolia*		
*Pyxine cocoes*		
*Roccella montagnei*		
*Cladonia coniocraea*	Beijing, China	Li et al. (2007)
*Melanelia sorediata*		
*Parmelia* sp.		
*Punctelia borreri*		
*Ramalina sinensis*		
*Xanthoria mandschurica*		
*Dermatocarpon miniatum*		
*Parmotrema* sp.	Hakgala Natural Reserve, Sri Lanka	Kannangara et al. (2009)
*Pseudocyphellaria* sp.		
*Usnea* sp.		
*Lobaria scrobiculata*	USA	Arnold et al. (2009)
*Nephroma arcticum*		
*Peltigera aphthosa*		
*Peltigera leucophlebia*		
*Peltigera malacea*		
*Peltigera neopolydactyla*		
*Peltigera scabrosa*	USA	Arnold et al. (2009)
*Umbilicaria mammulata*		
*Clavaroids* sp.	Bawang Mountain, China	Ding et al. (2009)
*Dermatocarpon* spp.	Chiricahua Mountains, USA	U’Ren et al. (2010)
*Flavopunctelia praesignis*		
*Punctelia hypoleucites*		
*Usnea hirta*		
*Pseudevernia intensa*		
*Xanthoparmelia viriduloumbrina*		
*Lecidea tessellata*		
*Physcia caesia*		
*Peltigera* spp.		
*Diploschistes muscorum*		
*Leptogium saturninum*	Zixi Mountain, China	Wu et al. (2011)
*Letharia vulpina*	–	Persoh and Rambold (2012)
*Pseudevernia intensa*	Chiricahua Mountains, Arizona, USA	Wijeratne et al. (2012)
*Cladonia leporina*	Florida, USA	Kamal et al. ()
*Cetraria islandica*	Laojun Mountain, China	Yuan et al. (2013)
*Lobaria retigera*	Mount Laojun, China	Dou et al. (2014)
*Xanthoparmelia* sp.	Zixisnan Mountain, China	Zhang et al. (2014)
*Cetrariella delisei*	Spitsbergen, Norway	Zhang et al. (2015)
*Cladonia borealis*		
*Cladonia arbuscula*		
*Cladonia pocillum*		
*Flavocetraria nivalis*	Spitsbergen, Norway	Zhang et al. (2015)
*Ochrolechia frigida*		
*Peltigera canina*		
*Parmelia caperata*	Similipal Biosphere Reserve, India	Padhi and Tayung (2015)
*Parmotrema reticulatum*	Kumaun Himalaya, India	Tripathi and Joshi (2015)
*Heterodermia flabellata*		
*Parmotrema praesorediosum*		
*Physcia dilatata*		
*Lethariella zahlbruckneri*	–	Li et al. (2015)
*Usnea* sp.	Botanical Garden, Sri Lanka	Samanthi et al. (2015)
*Cetraria islandica*	Laejun Mountain, China	Yuan et al. (2016)
*Usnea mutabilis*	Zixisnan Mountain, China	Yang et al. (2016)
*Everniastrum* sp.	–	Wang et al. (2012)
*Parmelinella wallichiana*		
*Usnea aciculifera*		
*Cladonia ochrochlora*		
*Peltigera elisabethae*		
*Hypogymnia hypotrypa*	China	Wang et al. (2016)
*Leptogium askotense*	Champawat, Uttarakhand, India	Suryanarayanan et al. (2017)
*Lobaria kurokawae*		
*Canoparmelia texana*		
*Parmotrema hababianum*		
*Parmotrema tinctorium*		
*Punctelia rudecta*		
*Usnea* sp.		
*Heterodermia diademata*		
*Heterodermia podocarpa*		
*Phaeophyscia hispidula*		
*Ramalina conduplicans*		

### Diversity of endolichenic fungi

Fossil lichen thalli from as early as the Lower Devonian had endolichenic fungal association in them (Honegger et al. 2013), suggesting that survival within lichen thallus is a successful strategy for fungi. Endolichenic fungi belong to the major lineages of *Ascomycota* to which the endophytic fungi of plants also belong (Arnold et al. 2009). They are distinct from the other fungal associates of lichen, namely the mycobiont (Lutzoni and Miadlikowska 2009) and the lichenicolous fungi associated with the lichens (Arnold et al. 2009). A molecular study by U’Ren et al. (2010) confirms that endolichenic fungi are a distinct ecological group and are not accidental colonisers of lichens. The endolichenic fungi are closely associated with the photobiont of the lichen. It is suggested that such an association could have led to the evolution of plant endophytes (Arnold et al. 2009). However, it appears that endolichenic fungi are distinct from the current endophytic fungi harboured by plants (U’Ren et al. 2012; Zhang et al. 2015). A comparison of endolichenic fungi at the genus level of some lichens and the endophytes of the trees on which these lichens were growing showed little overlap between them (Suryanarayanan et al. 2005). More data are required to confirm if some selection mechanism operates in the recruitment of endolichenic fungi by the lichens.

Like other heterotrophic associates of lichens, the endolichenic fungi depend on the photobiont for their nutrition. The photobiont species of a lichen species is known to vary (Ruprecht et al. 2014); however, it is not known if such a change in the photobiont partner affects the endolichenic fungal assemblage. This gains importance since such a low specificity in the selection of photobiont by the mycobiont in forming a lichen thallus widens the ecological amplitude enabling the lichen to colonise extreme environments (Muggia et al. 2014) and the diversity of endolichenic fungi is influenced by climate, host lineage and geographic isolation (U’Ren et al. 2012). In a recent study, Chagnon et al. (2016) conclude that endolichenic fungi are not strictly host specific and behave as generalists when compared with endophytes. Some endophytic fungi such as *Colletotrichum, Pestalotiopsis, Phomopsis* and *Xylaria* (Suryanarayanan et al. 2011; Govinda Rajulu et al. 2013; Sudhakara Reddy et al. 2016) also exhibit loose host affiliation and infect taxonomically unrelated plants. Different lichen species from different habitats have to be screened to determine the prevalence of such cosmopolitan endolichenic fungi. The species diversity of endolichenic fungi is hardly known although a pyrosequencing study showed that numerous fungi are present in lichen thalli (Bates et al. 2012). A multilocus phylogenetic analysis of the fungi of alpine rock lichens revealed the presence of fungal strains of new lineages within *Chaethothyriomycetes* and *Dothideomycetes* (Muggia et al. 2016). Recently, the use of molecular method has revealed the occurrence of 11 new species of lichen-forming fungi (Leavitt et al. 2016). Chagnon et al. (2016) argue that both culture-based and culture-independent studies have to be made to get a more complete picture of the diversity of endolichenic fungi. Additionally, lichen-enriched growth media could be used to isolate fastidious endolichenic fungi which fail to grow on normal growth media (Biosca et al. 2016). Some lichens infect already existing lichens and develop slowly as independent forms. A gene sequencing and fluorescence *in situ* hybridisation study reveals that there is a microbiome shift during such host–parasite lichen interaction (Wedin et al. 2016). The status of endolichenic fungal assemblages in such interactions is unknown. Similarly, the status of endolichenic fungi in marine and “borderline” lichens needs to be addressed. More information is needed on endolichenic fungi of different lichens growing in different environments to understand their pattern of distribution and host specificity and also to discern the influence of abiotic and biotic factors on their diversity.

### Role of endolichenic fungi in the lichen microbiome

When compared to endolichenic fungi, more information is available on the functional roles and biotechnological potential of bacterial associates of lichens (Grube and Berg 2009; Suzuki et al. 2016). These could serve as leads for addressing those of endolichenic fungi. Several bacterial communities are constantly associated with lichens and contribute to lichen symbiosis by (1) aiding in nutrient supply, (2) improving lichen’s resistance to abiotic and biotic stress, (3) detoxifying metabolites and (4) degrading older parts of the thallus (Grube et al. 2015). There is no information on the role of endolichenic fungi in the development and survival of lichens. While endophyte assemblage in plants is influenced by the age and chemistry of the host tissue (Arnold and Herre 2003; Suryanarayanan and Thennarasan 2004), it is not known if the age and chemical composition of a lichen influences the density of colonisation and species composition of its endolichenic fungi. Endophytes increase the ecological fitness of their host plants by making them more tolerant to abiotic stress including drought, salinity and high temperature (Sherameti et al. 2008; Sun et al. 2010). Such increased host fitness is achieved at least in a few cases by endophyte-mediated induction of stress-related host genes (Sherameti et al. 2008). Endophytes could also enhance a plant’s abiotic stress tolerance by altering its growth hormone metabolism (Khan et al. 2015). Lichens grow in harsh environments and exhibit wide tolerance to abiotic stressors; some of them survive prolonged exposure to desiccation, exposure to high (90°C) and low (−196°C) temperatures and high UV radiation (De Los Ríos et al. 2005; Grube and Berg 2009). A few of them like *Xanthoria elegans* survive exposure to simulated Martian atmosphere and real space conditions (De Vera et al. 2010). It would be of interest to know if endolichenic fungi, like endophytes of plants, aid in abiotic stress tolerance of their lichen hosts.

In some plants, the cost of harbouring endophytes (by way of loss of photosynthates) is compensated by the endophytes enhancing the host plant’s tolerance to certain biotic stressors. For instance, endophytes ward off insect pests (Estrada et al. 2015) and pathogens of their host plants (Arnold et al. 2003; Waqas et al. 2015). Endophyte infection upregulates numerous defence-related genes in the plant host (Mejía et al. 2014). Although it is known that during the early stage of interaction between the mycobiont and the photobiont in a lichen, several genes in both the partners are upregulated (Joneson et al. 2011), it is yet to be ascertained if, like the endophytes, the endolichenic fungi influence the gene regulation in their lichen hosts.

The endolichenic microbiome elaborates many antimicrobial chemicals. A metagenomic and culture-based approach showed that the lichen *Lobaria pulmonaria* harbours a bacterial community which elaborates many microbial antagonistic agents (Cernava et al. 2015). With reference to endolichenic fungi, of the 62 isolates screened for production of antialgal and antifungal metabolites, 45 and 37 isolates exhibited antialgal and antifungal activity, respectively, and 30 isolates showed both antialgal and antifungal activity (Suryanarayanan et al. 2017). It would be of interest to know the interaction between such antialgal metabolite producing endolichenic fungi and the photobiont of the lichen. Interestingly, the removal of secondary metabolites from the lichen thallus increases invertebrate abundance in them (Asplund et al. 2015). These studies suggest that the bacteria and fungi inside lichen thallus could influence its biotic stress tolerance ability. A detailed study of the metabolite spectrum of the various endolichenic fungi is needed to confirm their influence on alteration of a lichen’s fitness.

Lichens are an attractive source of unique secondary metabolites (Kumar et al. 2014); so far, more than 1000 secondary metabolites have been extracted from lichens found growing from the Arctic to the tropics (Stocker-Wörgötter 2008). About 0.1–5% of the dry weight of lichen thallus is composed of secondary metabolites derived from the acetyl polymalonyl, mevalonic and shikimate pathways (Stocker-Wörgötter 2008). Most of these metabolites are of fungal origin (Stocker-Wörgötter 2008; Abdel-Hameed et al. 2016) and perform ecological functions including regulation of symbiosis; many of them possess various technologically attractive bioactivities (Bačkorová et al. 2012). Apart from the lichens, the endolichenic fungi themselves, like the plant endophytes (Suryanarayanan et al. 2009; Kaushik et al. 2014; Chen et al. 2016), are a good source of novel bioactive molecules (Chang et al. 2015; Kellogg and Raja 2016). Following this, it would be worthwhile screening endolichenic fungi for their ability to produce novel metabolites exhibiting specific biological activities.

Another aspect to be unravelled is the interactions between endolichenic fungi and the host lichen and between endolichenic fungi and other groups of organisms that lichens support. Many lichens elaborate antifungal metabolites (Basile et al. 2015) and hence a lichen’s endolichenic fungal community should be insensitive to these metabolites or be able to detoxify them. It could be assumed that only such fungi would constitute the endolichenic fungal assemblage of a given lichen. Such endolichenic fungi could be screened for their ability to biotransform metabolites to produce novel pharmaceuticals and to degrade recalcitrant chemicals (Wang and Dai 2011). It is known that infection of the Alpine lichen *Solorina crocea* by fungi leads to alteration of its bacterial community (Grube et al. 2012). Similarly, production and detoxification of antimicrobial compounds by endolichenic fungi *in vivo* could lead to interspecific competition ultimately determining the species composition of bacterial and fungal assemblages within the lichen thallus (Suryanarayanan et al. 2017). Given the fact that endolichenic fungi also produce antimicrobial metabolites (Suryanarayanan et al. 2017), competition and cometabolism among the lichen microbiome need to be studied using modern techniques such as isotopologue profiling for understanding the cross talk between the various constituents of the lichen microbiome (Götz et al. 2010). Analysis at the gene level of endolichenic fungi would help in understanding their contribution to the secondary metabolite profile of lichens and their possible function in the lichen microbiome. Like the endophytes of plants, the endolichenic fungi may have the ability to produce an array of secondary metabolites due to the presence of novel genes; such novel genes could have evolved as a consequence, a selection pressure exerted by interactions between different entities of the lichen microbiome. Generally, a majority of the natural products genes of fungi are silent under normal culture conditions. Several methods including genomic mining, Proteomic Investigation of Secondary Metabolism, genetic manipulation of biosynthetic pathway genes and the use of epigenetic modifiers are in vogue to induce these cryptic secondary metabolite gene clusters (Lim et al. 2012). Mining endolichenic fungi for novel secondary metabolites using these modern methodologies could yield pharmaceutically important products.

As pioneer colonisers of rocks, lichens play a role in biotic weathering of rocks and other biogeochemical processes such as soil formation and nutrient cycling. Lichens also accumulate heavy metals and radionuclides (Gadd 2007). Although fungi are known to weather rocks by producing carbonic and other acids (Hoffland et al. 2004) and scavenge heavy metals and tolerate radionuclides (Cordero and Casadevall 2017), the contribution of endolichenic fungi to these phenomena exhibited by lichens is not known.

Endophytic fungi of plants are a novel source of several enzymes including biomass degrading and pharmaceutical enzymes (Suryanarayanan et al. 2012). Many of these fungi produce novel chitinases and chitosanses (Govinda Rajulu et al. 2011), proteases (Thirunavukkarasu et al. 2017), cellulase, laccase, lipase and pectinase (Govinda Rajulu et al. 2013). Endophytic fungi of marine plants produce salt-tolerant xylanases and xylosidases (Thirunavukkarasu et al. 2015). Endolichenic fungi have not been screened for the production of such enzymes. Bacteria associated with lichens degrade parts of lichen thalli to aid biomass mobilisation (Grube and Berg 2009). Endolichenic fungi may also be involved in degrading dead parts of lichen thalli (epinecral layers of lichen thalli).

Lichens represent an evolutionarily successful mutualism and support a microbiome constituted by different microorganisms. The interactions between these microbes and between the microbes and the environment are complex resulting in the creation of a unique microenvironment within the lichen thallus. Microbes in such a milieu could be a good source of biotechnologically important metabolites. In this context, the endolichenic fungi are the least studied of the lichen microbiome. Endolichenic fungal association could have evolved along with the lichens as has been construed for bacterial association with lichens (Grube and Berg 2009). With very few lichen species studied endolichenic fungi, it is reasonable to expect many hitherto undescribed fungal species to be present in this ecological group of fungi (Muggia et al. 2016). It is also possible that the endolichenic fungi house many novel genes whose products could be exploited technologically. This gains further importance when we consider the fact that the lichen diversity itself is not be fully known (Boch et al. 2013). It is necessary to understand the role of endolichenic fungi to appreciate the role of lichens as determinants of ecological processes which itself is often overlooked (Asplund and Wardle 2016).
